# Isolating the Role of Bone Lacunar Morphology on Static and Fatigue Fracture Progression through Numerical Simulations

**DOI:** 10.3390/ma16051931

**Published:** 2023-02-26

**Authors:** Federica Buccino, Francesco Cervellera, Marta Ghidini, Riccardo Marini, Sara Bagherifard, Laura Maria Vergani

**Affiliations:** Department of Mechanical Engineering (DMEC), Politecnico di Milano, Via La Masa 1, 20156 Milano, Italy

**Keywords:** bone damage, lacunar morphology, XFEM, fatigue analysis, fracture initiation

## Abstract

Currently, the onset of bone damage and the interaction of cracks with the surrounding micro-architecture are still black boxes. With the motivation to address this issue, our research targets isolating lacunar morphological and densitometric effects on crack advancement under both static and cyclic loading conditions by implementing static extended finite element models (XFEM) and fatigue analyses. The effect of lacunar pathological alterations on damage initiation and progression is evaluated; the results indicate that high lacunar density considerably reduces the mechanical strength of the specimens, resulting as the most influencing parameter among the studied ones. Lacunar size has a lower effect on mechanical strength, reducing it by 2%. Additionally, specific lacunar alignments play a key role in deviating the crack path, eventually slowing its progression. This could shed some light on evaluating the effects of lacunar alterations on fracture evolution in the presence of pathologies.

## 1. Introduction

Bone fracture prediction and prevention represent core challenges to be actively addressed in an elderly society, where the occurrence of skeletal aging and genetic and metabolic bone diseases is dramatically increasing [1,2]. All these concurrent factors lead to an intensification of the fracture risk, severely affecting bone mechanical integrity [3]. The social and clinical relevance of the mentioned issue is combined with an added complexity intrinsically linked to the hierarchical bone structure, meaning that there are discrete characteristic structural features that differ at diverse length scales.

The application of an engineering fracture mechanics approach has been of essential aid when clarifying macro- and meso-scale bone deformation and fracture [4,5,6], extrapolating quantitative relationships between toughness and crack extension (R-curves) [7,8]. However, while at larger scales, the synergy of clinical diagnostic tools and engineering approaches [9] has been able to provide quantitative and qualitative information on bone characteristics, at present, only preliminary and often not validated strategies are available to analyze bone damage at the micro-scale [2,10,11,12].

At the micro-scale, osteocytes that orchestrate bone remodeling processes are embedded in a dense network of ellipsoidal porosities, i.e., lacunae, the connectivity, shape, density, size, and orientation of which vary during aging, and in the presence of bone pathologies [13,14,15]. As an example, in the widespread osteopenia (OP) disease, characterized by a loss in bone mineral density, lacunae show a large size and roundish shape and tend to be aligned with respect to the externally applied load. An opposite trend is visible in osteopetrosis (PET), also known as marble bone disease, which induces an increase in bone mineral density and alters bone micro-morphology, leading to smaller roundish lacunae, randomly arranged in the three-dimensional space. Regarding lacunae–crack cross-relationships, in the actual research landscape, lacunae seem to play an antithetical mechanical role, affecting both the strength and toughness of the bone. On the one hand, lacunae represent stress and strain intensification sites, with a local average strain 1.5–4.5 higher than the remote strain applied to the surrounding tissue [16,17,18]. On the other hand, considering bone is a damage-tolerant material, lacunae are able to deviate from the crack path, slowing down damage progression.

In order to shed some light on this delicate mechanobiological phenomenon, the use of advanced computational tools is preferred to extensive experimental campaigns on human or animal samples, in accordance with the 3R principle, which proposes the replacement, reduction, and refinement of animal experiments in favor of tests on surgical wastes and on in silico models [19,20].

In this scenario, extended finite element models (XFEM) appear as promising tools for their ability to deal with discontinuous problems, such as initiation and propagation of cracks, without needing to re-adapt the mesh with the discontinuity at each increment of the analysis, which happens with traditional finite difference methods. Indeed, classical finite element methods could be employed to evaluate stress–strain alterations due to eventual discontinuities such as bone micro-scale features [13], but lack in evaluating crack advancement phenomena. In this regard, several authors [21,22] compared two XFEM modeling techniques, i.e., Cohesive Zone Modelling (CZM) and Linear Elastic Fracture Mechanics (LEFM) used to analyze the crack path across the osteon cortical structure. CZM, which uses traction separation law to describe how the elements gradually deteriorate, is reported to produce comparable results with respect to LEFM, which exploits Virtual Crack Closure Techniques (VCCT) to model crack propagation. To deal with the effects of meso- and micro-scale porosities and their interaction with cracks, preliminary two-dimensional (2D) and three-dimensional (3D) models have been implemented. Regarding the 2D XFEM models, huge interest is devoted to the analysis of the multi-toughening mechanism of the osteonic micro-structure and to the study of lacunar arrangements [23,24]. Lacunar voids are modeled as circles or ellipses, and cracks are inserted a priori in the computational model.

The evident limitations induced by shape simplification and 2D crack progression are partially overcome by recent attempts in 3D XFEM modeling of bone crack propagation, with the goal of capturing the spatial evolution of damage. However, these approaches are limited to the macro-scale, where the vigorously debated effects of porosities are entirely neglected [25,26].

In this context, the present work aims to overcome the discussed restrictions in bone lacunar shape simplifications, bi-dimensional crack evolution, and pre-defined crack initiation sites by isolating real human lacunar morphologies to elucidate their influence on static and fatigue fracture progression. The purpose of analyzing both static and cyclic scenarios is directly linked to the stance and swing phase of human gait, which could potentially have an effect on, as an example, the lacunar disposition of lower limbs [27,28]. Indeed, the effects of lacunar density, alignment, and size are separately investigated to extrapolate critical parameters for crack advancement under both loading conditions. Specifically, lacunar morpho-densitometric features are isolated from the bone context, and their effects on damage initiation and progression are numerically studied in AISI316L cubic specimens. This will allow for evidencing eventual toughening and weakening patterns happening independently from the material choice and directly linked to the pore arrangement and shape.

## 2. Materials and Methods

### 2.1. Specimen Design

**Figure 1:**
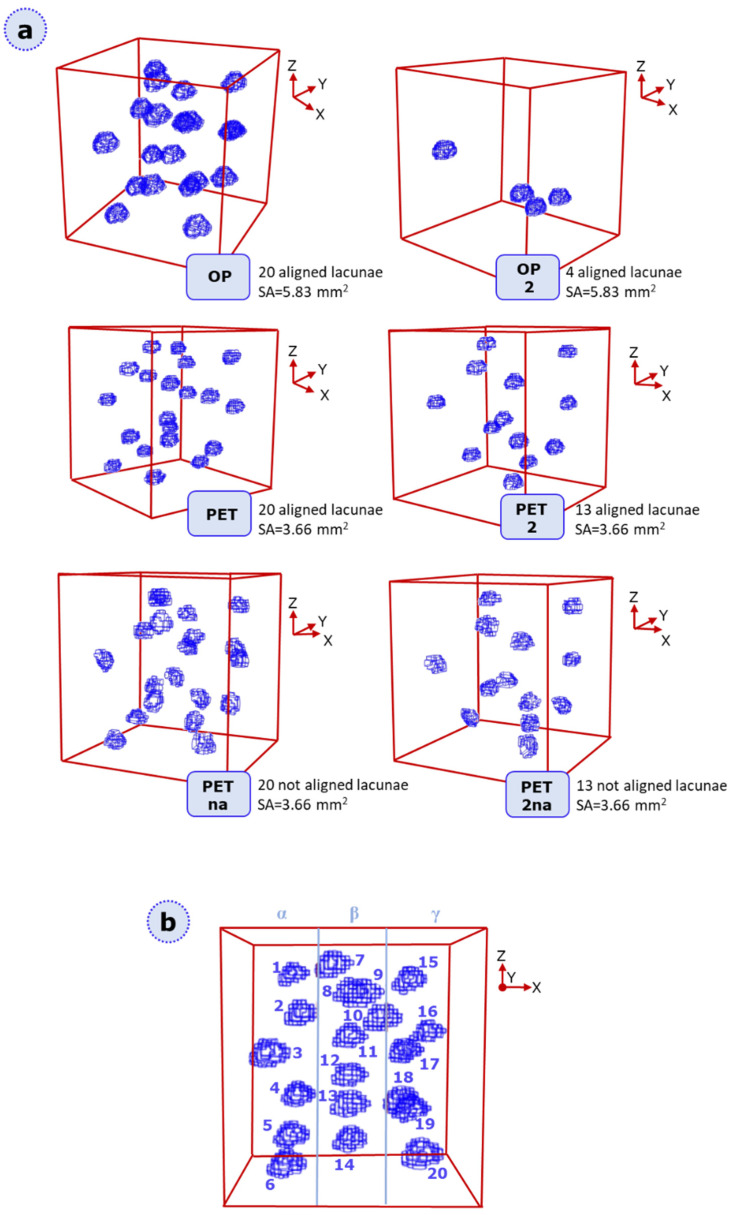
(**a**) overview of the six specimen categories: OP, OP2, PET, PET2, PETna, PET2na. The number 2 refers to a change in density, while “na” is related to the misalignment of the lacunae. For each geometry, we report the number of embedded lacunae and the surface area of each lacuna. (**b**) Model equal subdivision in three different zones of the same dimensions, i.e., α, β, γ. The sample is a cubic specimen of 8 mm side length. This nomenclature allows for critical regions for damage occurrence to be better located. The exemplificative category represented here is OP; lacunae of each tested category are identified in an analogous way, following a top-to-bottom ascending enumeration for each partition. The same lacunar classification process is repeated for all the other categories.

Six cubic specimens containing lacunar-like voids were considered in this work. Lacunar geometries and distributions were designed based on observations in human pathological bones, as detailed in van Hove et al. [29]. The nomenclature of specimens consists of the following (Figure 1a): OP or PET that refers, respectively, to osteopenic-inspired structure and osteopetrotic-inspired structure. The number “2” after those labels refers to a change in lacunar density; the letters “na” (not aligned) identify a change in lacunar orientation, i.e., misalignment. With these designs, the effects of both morphological and densitometric lacunar parameters can be analyzed on crack initiation and progression; furthermore, density, size, and alignment/orientation with respect to the applied load are deemed as remarkable factors for altering crack propagation.

The designed specimens (see Figure 1a) of 8 mm side length were numerically analyzed under static and fatigue loading conditions. A fictitious partitioning in planes was proposed for each specimen in order to proceed with lacuna numbering following a top-bottom order for each identified sub-region (Figure 1b).

After model realization and mesh sensitivity analysis, a shrink wrap mesh of C3D8R–3D continuum elements, with reduced integration and characteristic size of 0.14 and 0.16 mm, was adopted in Hypermesh 2019 software. OP2 is the only geometry meshed with 0.16 mm elements due to the low number of lacunar discontinuities that permit coarser element sizes. The mesh size is inversely linked to the computational costs; indeed, increasing the element size reduces the computational costs. This, however, comes with a reduction in the accuracy of the results. In our specific case, the choice of 0.14–0.16 mm mesh size represents the best compromise. Indeed, if we compare the results obtained with a 0.12 mm mesh size, our increase in mesh size up to 0.16 mm is not dramatic because the reported difference between the selected output control values (reaction forces) is around 0.5%, which is realistically negligible. The minimum Jacobian value was 0.8 for all models. A jacobian value of 1 was avoided since a complete discretization with cube-shaped elements risks altering too much the original roundness of the lacunae in the geometries: a jacobian of 0.8 is considered a good compromise between element shape and fidelity on the original geometries.

### 2.2. Static XFEM Analysis

The adopted XFEM tool is a powerful strategy implemented in Abaqus 2019 software (Abaqus CAE, Dassault Systèmes, France, 2019) to study the onset and propagation of cracking in the present quasi-static problem. Theorized in 1999 [30] and based on the partition of unity property [31], it is an extension of the conventional FEM by enriching the elements with extra degrees of freedom (DOFs), as described in Equation (1). Each addendum of Equation (1) refers to a different contribution: the first one is related to the standard FEM displacement field; the second one is linked to the enrichment due to the element cut by a discontinuity; and the third one corresponds to the enrichment at the crack tip. **u** is the total displacement matrix, N_i_ are the shape functions, **u_i_** is the nodal displacement, and H(x) is the Heaviside step-like function. Not all the nodes in a defined enriched region are actually enriched by additional DOFs; only the elements divided by a singularity have their respective nodes characterized by additional degrees of freedom (**a**_i_ and **b**_i,α_ in Equation (1)). The term B_α_ refers to elastic-asymptotic crack-tip functions describing the crack front.

(1) $$u=\underset{i\in N}{\sum }N_{i}\left(x\right)u_{i}+\underset{i\in N_{\mathrm{cut}}}{\sum }N_{i}\left(x\right)H\left(x\right)a_{i}+\underset{i\in N_{\mathrm{front}}}{\sum }\underset{\alpha }{\sum }N_{i}\left(x\right)B_{\alpha }\left(x\right)b_{i,\alpha }$$

Here we adopted the CZM approach [32,33] to study crack evolution. It is based on the idea that complete fracture is reached through a progressive separation of the two crack interfaces in a confined, small zone ahead of the crack tip. In this scenario, Hillerborg et al. [34] proposed a strategy to alleviate the mesh dependency of the problem by considering a stress–displacement response after damage initiation. The singularity at the crack tip is considered unphysical, and the stress distribution in the process zone is unknown and cannot be generally measured. Therefore, the stress does not depend on the distance from the crack tip, but it is related to a fictitious opening (displacement—u). The proposed nodal displacement governing equation will not, hence, consider the third addendum related to the enrichment terms of the crack front. The constitutive response of the elements upon damage is a traction–separation law (TSL) that requires an initiation threshold stress and a damage evolution parameter [35] in the form of displacement at failure (DAF, u(f)) or (cohesive) fracture energy (Γ).

For elucidating damage occurrence in the geometries presented in Figure 1, we defined a static XFEM analysis; the material definition is provided by assuming a linear-elastic behavior. For this study, since we aimed at isolating lacunar morpho-densitometric features from the original bone context, we considered AISI 316L material characteristics; indeed, this stainless steel is adopted in an ongoing parallel study on printability via laser powder bed fusion of the described geometries. The specific choice of AISI 316L is directly linked to its extensive adoption in the production of implants and biomedical devices. For XFEM static analyses, Young modulus (E) was set to 156,360 MPa, the ultimate tensile strength (UTS) corresponds to 605 MPa, and the strain at break corresponds to 34.7%. Poisson’s ratio of 0.3 is considered [36]. Future studies are planned to deepen the effects of lacunar cavities in other biomedical materials, such as titanium.

TSL requires defining damage parameters in the material model in terms of damage initiation and evolution. Thus, we employed a stress-based damage initiation criterion defined by the maximum principal stress (MAXPS). This criterion allows crack propagation across the elements in a solution-dependent way: crack propagation occurs perpendicularly to the maximum principal stress, and the discontinuity allows the crack to change plane and direction during propagation. Therefore, the crack path growth is not pre-defined along a pre-selected direction [37,38,39]. Finite element analysis in the presence of continuum elements is based on the virtual work statement that considers Cauchy “true” stresses; therefore, the engineering UTS value is converted into the “true” UTS value [39] that is employed as MAXPS, i.e., 756 MPa in our case. The minimum tolerance related to the accuracy of damage initiation value for the performed numerical analysis results in being 0.1, and the maximum is 0.2; tolerances below this value may lead to converging simulations with no crack propagation.

Concerning damage evolution, we adopted the simplification of considering a linear traction-separation law. Several studies have worked on modeling traction–separation [40,41], such as the one proposed by Tvergaard and Hutchinson [42] that best fits elastoplastic stress–strain curves. However, it has been confirmed that even if the selected material is ductile, a TSL model typically used to model brittle failure can be employed without considerably altering crack initiation and propagation [42,43,44]. Linear degradation of the TLS stiffness matrix is displacement-controlled through the value set for displacement at failure (DAF), which is mesh dependent. It is strictly related to the cohesive fracture energy Γ(c). Our hypothesis of considering Γ(c) equal to the fracture energy G(c) calculated as the area under the true stress–strain curve is typically considered a good approximation. However, there is some uncertainty about the exact way to evaluate the damage evolution parameter within the XFEM-TSL environment. Indeed, it is also suggested [35] to compute Γ(c) through Equation (2), where L refers to the characteristic length of an element and is introduced to reduce the computational time, and ε(n.o.) refers to the strain at the onset of necking.

(2) $$\Gamma_{c}=L\int_{\varepsilon_{n.o.}}^{\varepsilon_{f}}\sigma d\epsilon$$

It is particularly complex to define a precise value of fracture energy, as demonstrated in the Results Section 3.1, to be employed in XFEM analyses on porous arrangements; a recent work [37] shows how a certain range of fracture energies could be adopted to simulate fracture evolution. Here, two different values were adopted, one obtained by considering the entire area under the true stress–strain curve (high fracture energy) and one evaluated by just considering the area under the necking region (low fracture energy). The input data for DAF are 0.0668–0.0217 mm for the 0.14 mm meshed models and 0.0764–0.0247 mm for the 0.16 mm meshed models. Since MAXPS and UTS should coincide and correspond to the same strain value, for the considered numerical campaign on lacunar-embedded geometries, it is appropriate to employ a low fracture energy related only to the necking region (see Supplementary Material); however, some adaptation and cohesive fracture energy calibrations could be considered when altering the geometrical characteristics of the porosities.

Regarding the convergence parameters, we mainly focused on viscous regularization [25,45,46,47,48,49], set to 10^−5^, and automatic stabilization. This latter was set to adaptive, with a damping factor based on the dissipated energy fraction; the initial dissipated energy fraction and its accuracy tolerance were set as default, i.e., 2 × 10^−4^ and 0.5, respectively. Post-analysis comparisons to assess whether these artificial parameters have a major influence on the simulations were carried out. Specifically, we checked that the ratios of dissipated viscous energy (ALLVD) to recoverable strain energy (ALLSE) and energy dissipated by viscous damping (ALLSD) to the total strain energy (ALLIE) were not higher than 2% during the analysis.

**Figure 2:**
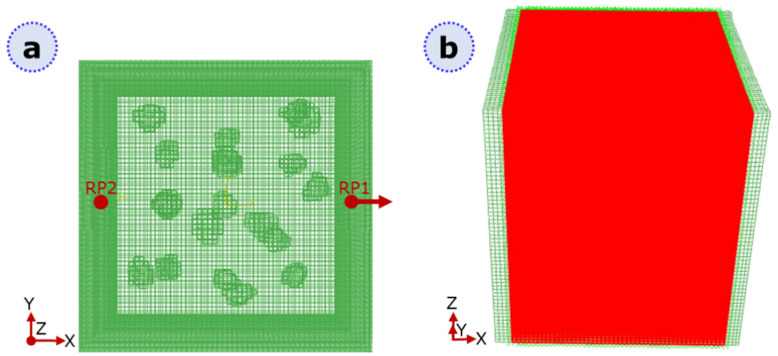
(**a**) Schematic of the boundary conditions adopted in the model: the surface coupled with reference point 1 (RP1) is pulled into displacement-controlled traction, whereas the surface coupled with RP2 is fully constrained. (**b**) The crack domain is highlighted in red; the surfaces mentioned in (**a**) are left out from the domain since constrained features may alter the stress distribution due to boundary effects.

Attention should be paid tp defining proper boundary conditions, crack domain, and outputs. Figure 2 shows how the boundary conditions (Figure 2a) and the crack domain (Figure 2b) are selected for static analysis. Displacement-controlled traction occurs through a linear ramp that follows the automatic time increments, the final value of which is set to 0.5 mm. In order to effectively observe the damage progression in the results, damage variables must be defined as outputs of the analysis. Specifically, the variable STATUSXFEM is an indicator of the cohesive property loss in an element, assuming values in the range from zero to 1, i.e., from no loss (STATUSXFEM = 0) to complete failure of the element (STATUSXFEM = 1 cohesive stiffness degradation). All the values in between refer to a partially damaged element.

In the last phase of output post-processing, a color-identifier algorithm in MATLAB was adopted to evaluate the percentage of completely/partially broken elements, i.e., STATUSXFEM = 1, in each model.

### 2.3. Fatigue Analysis

FeSafe 2019 software (SIMULIA, Dassault Systèmes, France, 2019) was employed to study the fatigue behavior of all the aforementioned geometries when subjected to cyclic loads. The goal was to obtain an independent indication of the most critical lacunae by looking at the number of cycles required to nucleate cracks. Therefore, fatigue simulations were conducted by using the Brown–Miller strain-based algorithm typical of ductile materials. Strain-based simulations provide the total number of cycles required to initiate crack propagation at every given point in the specimen. Regarding boundary conditions and loading characteristics, specimens were subjected to uniaxial sinusoidal tensile traction load applied on the side surface. The stress ratio was set equal to 0.1, the scale (alternate stress) corresponded to 148 MPa, and the offset (mean stress) was 180 MPa. These values were chosen considering the fatigue behavior of the selected material [50]. No pre-defined crack initiation site was defined. The material was considered isotropic, specifically because we would like to isolate lacunar-like features and evaluate their effect in the presence of fatigue in a material that is completely different from bone.

## 3. Results

### 3.1. Effects of Damage Evolution Parameters on Crack Initiation

As an initial core point, we checked the effects of diverse damage evolution parameters on the static strength of lacuna-embedded geometries. Since no major differences can be appreciated for what concerns damage patterns, we mainly focused on force (i.e., reaction force)–displacement curves and traction–separation curves. It is particularly intricate to define a precise value of fracture energy to be employed in XFEM analyses on porous arrangements; a recent work [37] shows how even a certain range of fracture energies could be adopted to simulate fracture evolution.

**Figure 3:**
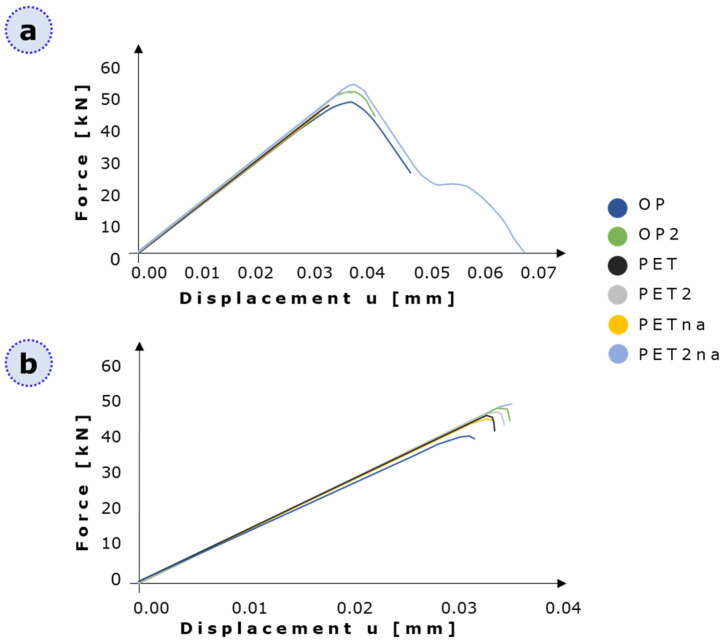
Force–displacement curves for all the tested geometries with (**a**) the higher and the lower (**b**) value of DAF.

As highlighted in Figure 3, the outputs related to the analysis with lower DAF, in general, show an increasing trend of peak forces when increasing the geometry porosity. Table S1 in the Supplementary Materials reports the corresponding displacement in the proximity of the reaction force peaks related to the simulations conducted with lower DAF.

High energy curve peaks are present at the same value of displacement; this is an unrealistic result for models containing drastic differences in lacunar density and orientation. On the other hand, low energy curves show a clearer distinction between the peak positions of the different geometries, which is likely to be more accurate.

**Figure 4:**
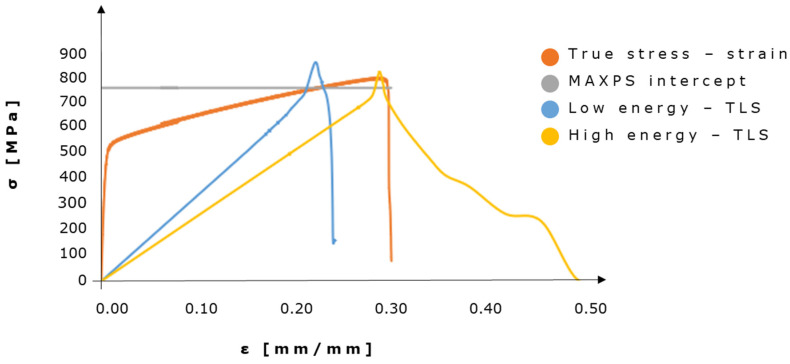
Stress–strain curves: true stress–strain, MAXPS intercept, curves at low and high energy TSL. The true experimental stress–strain curve is derived from AISI 316L datasheet [36].

Regarding traction–separation curves, we reported TSL curves for elements that experience full discontinuity (i.e., STATUSXFEM = 0). Figure 4 shows the comparison of two TSL curves and the true experimental stress–strain curve. In order to obtain the true strain values for the traction-separation curves, displacement was divided by the characteristic length of the element for each model.

When comparing TSL graphs obtained from two completely broken elements belonging to different fracture energy models, the overall shape of the curves changes significantly. The low-energy TSL curve qualitatively fits the failure zone more accurately than the high-energy curve. Indeed, as in the true stress–strain curve, the low energy curve sharply drops after having reached the MAXPS (Figure 4). It has to be noted that the initial regions of the curves cannot be compared since TSL is defined as linear; hence the maximum principal stress value will be reached through a linear ramp instead of a “trapezoidal” one. On the other hand, the low energy curve reaches a value of strain that is 30% higher than the theoretical DAF of 0.0217 (0.0247 for OP2), while the high energy curve accurately reaches the imposed DAF. Nonetheless, this inaccuracy can be likely explained by the imposed tolerance, which is also responsible for a 10–15% increase in both high- and low-energy TSL curve peaks with respect to the MAXPS of 756 MPa.

Since MAXPS and UTS should coincide and correspond to the same strain value, it can be concluded that for the considered numerical campaign on lacunar-embedded geometries, it is appropriate to employ a low fracture energy related only to the necking region; however, some adaptation and cohesive fracture energy calibrations could be considered when altering the geometrical characteristics of the porosities.

### 3.2. XFEM Static Analysis of Lacuna-Embedded Geometries

**Figure 5:**
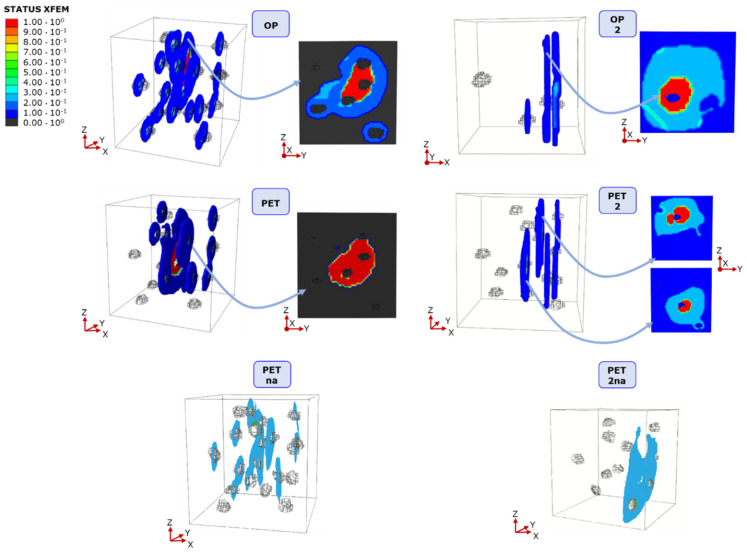
STATUSXFEM distribution in the six lacunar-embedding geometries after the application of displacement-controlled traction. All the non-red elements experience partial damage, while red elements correspond to complete failure. For OP, OP2, PET, and PET2, characterized by completely failed elements, the critical plane is reported close to the 3D view.

With the aim of comparing damage characteristics in each lacuna-embedded specimen, we report a visualization output for each specimen category for simulations at lower DAF values in Figure 5. STATUSXFEM values are shown for the ultimate convergence increment, allowing for the check of the element’s cohesive properties loss and, therefore, for the evaluation of damage patterns.

The precise percentage of completely/partially broken elements, i.e., STATUSXFEM = 1, is computed for each specimen category. These percentages are related to specific surfaces selected in a post-analysis study. The surfaces with the highest number of completely/partially broken elements are identified, and their distance from the traction surface (traction is occurring in the positive x direction of Figure 5) is underlined. OP specimen shows a percentage of failed elements of 5.71%, which is mainly located 4 mm far from the traction surface. Comparable results regarding broken elements are visible in OP2, where 6.86% of red elements are located at a distance of 2.3 mm from the traction surface. All the non-red elements experience partial damage, and their related percentage is below 30% (dark blue color). Regarding the PET category, 11.04% of elements were identified as failed 4 mm away from the traction surface, whereas 30% of non-red elements were subjected to partial damage. Higher critical elements are visible in PETna and PET2na specimens (40% and 42.05%, respectively), with a percentage of damaged elements corresponding to 25% and 40%. Two critical planes were identified in PET2 with 2.42% and 5.62% broken elements located at distances of 4 mm and 2.8 mm from the traction surface, respectively. For PET2, the damaged elements are about 33%.

Additionally, Table S2 in the Supplementary Materials identifies, for each specimen category, lacunae that are sites of damage initiation.

### 3.3. Fatigue Analysis of Lacuna-Embedded Geometries

**Figure 6:**
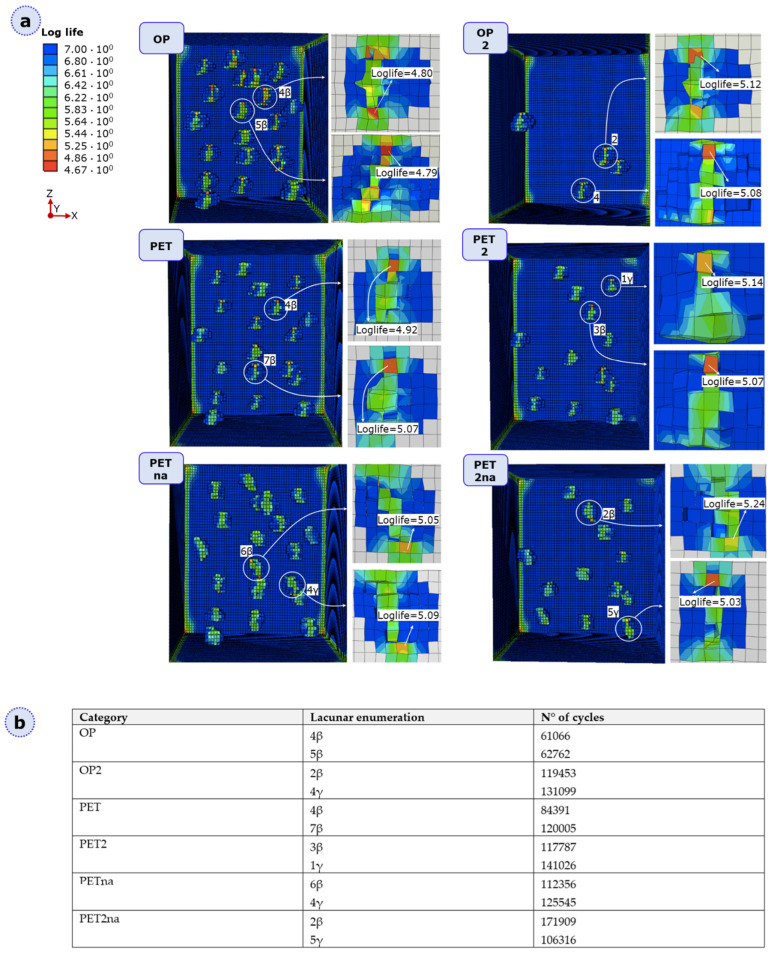
(**a**) Schematic of the numerical results obtained under fatigue testing for the six categories. The log-life value is reported for the two most critical lacunae in each geometry. (**b**) Number of cycles to damage initiation. For each geometry, two most critical lacunae are reported, representing initial crack nucleation and potential crack progression sites.

With the analogous purpose of locating the most critical lacunar network for damage initiation but under fatigue loading conditions, FeSafe high cycle fatigue analyses were carried out. For each geometry, we considered the number of cycles to crack initiation (log-life) as a suitable parameter to assess the critical sites for crack onset. Figure 6a reports the most critical lacunae in each geometry, with a specific reference to the log-life. In 80% of the cases, the β region appears as the most prominent zone for crack initiation, with damage appearing at a lower number of cycles in the OP configuration with respect to other geometries (Figure 6b).

## 4. Discussion

With the aim of addressing the intimate cross-talks existing between human bone lacunae and micro-cracks, our approach started by isolating lacunar morphology in osteopenic and osteopetrosic subjects. This choice specifically resides in different features exhibited by OP and PET bone micro-scale architecture, resulting in opposite effects on bone mineral density and strength. Computational XFEM static and fatigue analyses were conducted on six 3D porous geometries, succeeding in evaluating and localizing critical damage initiation and progression sites. In detail, we deepened the separate effects of lacunar density, size, and orientation on the mechanical strength of bone-inspired AISI 316L samples. Furthermore, we considered the realistic 3D shape of lacunae, and we analyzed damage initiation sites in the absence of pre-cracking, overcoming the simplifications highlighted in the current state-of-the-art when schematizing lacunae as perfect ellipses or adopting fictitious crack onset sites to speed up the convergence.

Regarding the number of failed elements in XFEM simulations, the OP specimen shows a percentage of failed elements of 5.71%, which is mainly located 4 mm far from the traction surface and is lower than the PET case (Figure 5). All the elements that are not depicted in red or light blue (dark blue and black) experience less than a 20% reduction in cohesive properties. For PET, twice the number of PET failed elements were identified 4 mm away from the traction surface.

**Figure 7:**
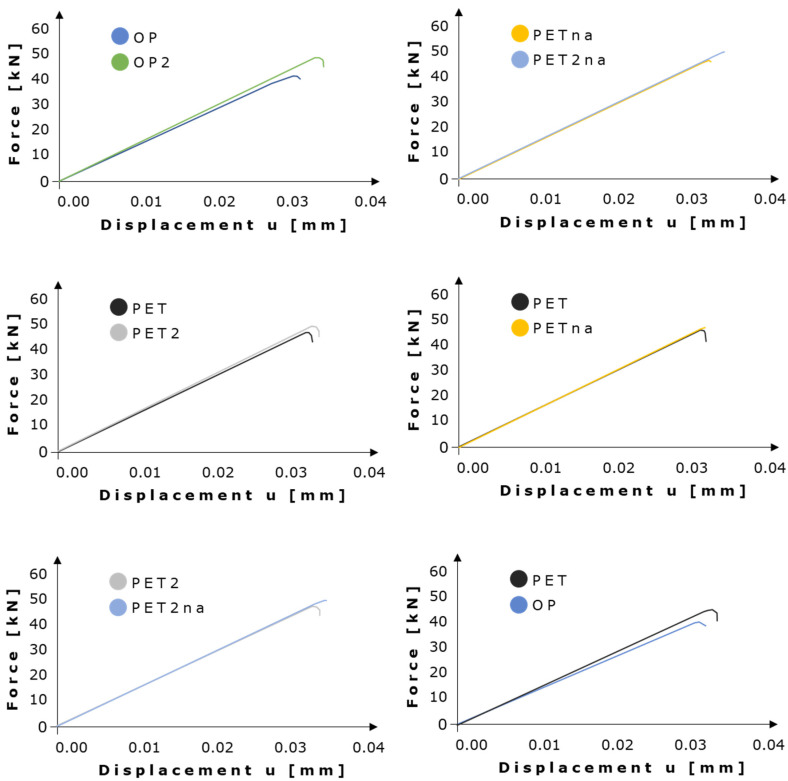
Investigation of the lacunar characteristics effects on mechanical strength, showing the force–displacement curves extrapolated from static XFEM analyses.

After properly tuning the computational parameters and quantifying their effect in static XFEM analyses, we focused on the detailed investigation of lacunar features on the specimen’s mechanical strength by referring to the force–displacement curves (Figure 7). Interesting parallels could be performed with behavior detected in human bones that are subjected to both static and fatigue loads.

The predominant parameter affecting the loss of mechanical strength is an increase in lacunar density, with an exception represented by PETna [13]. This model, however, is the only one characterized by a single damaged plane with a loss in cohesive properties of around 40% (Figure 7). Therefore, this loss in cohesive strength is not enough to cause an overall critical reduction in the mechanical strength of the model; hence, partial damage extended by 20% was not found to be critical for the specimen strength. However, we believe that the formation of secondary partially damaged regions, as shown in all the other lacunar-embedded categories, is a more realistic condition since lacunae alone should act mainly as stress raisers (as highlighted in human bone damage [13]), resulting in damaged elements around them (Figure 6a). Therefore, OP2 with four lacunae appears as the most resistant specimen; by increasing the lacunar number to 13 (therefore increasing the porosity), PET2 shows a reduction of 1.8% in the displacement at failure. An additional drop of 9% is visible in the 20 lacunae specimen, i.e., OP. When comparing PET2 and OP, this value becomes 8.1% with a 35% rise in the lacunar number.

The lacunar size is responsible for the limited reduction of about 2% in mechanical strength (Figure 7). Indeed, the overall lacunar surface area in the case of OP2 is 22 mm^2^, the one related to PET2 is 49.4 mm^2^, and the one linked to OP is 110 mm^2^. Even if the ratio between OP2 and PET2 surface area and the one between PET2 and OP is quite the same, the actual magnitude of these values plays the main role; that is, passing from OP2 to PET2 means increasing the overall lacunar surface area by 27.4 mm^2^, whereas passing from OP2 to OP this value rises to 88 mm^2^ and from PET2 to OP it becomes 60.6 mm^2^. We, therefore, believe that variations in lacunar size and density are strongly interconnected since changing one or both of them still has the same effect of altering the total porosity of the models. This consideration is also supported by the fact that PET and OP have the same lacunar density but a different lacunar size, and PET fails at higher traction values with respect to OP. As mentioned, the lacunar surface area of OP is 110 mm^2^, whereas the one related to PET is 76 mm^2^; therefore, the overall OP porosity is higher with respect to the one of PET.

The influence of a random lacunar alignment on mechanical strength is, instead, less evident, starting from the aforementioned considerations regarding the predicted traction at failure for PET2na. Moreover, neither PET2na nor PETna experiences heavily damaged elements (Table S2, Supplementary Materials); this observation can be justified by considering that, in the case of PETna, the misalignment of the lacunae is able to split the crack path, hence demanding more energy to produce multiple fracture surfaces, which realistically happens in human bone micro-damage. By relating PET2 and PET2na, we are led to think that misalignment of the lacunae leads to a slower damage progression.

Regarding the influence of lacunar morphological and densitometric parameters on fatigue resistance, we specifically discussed the number of cycles required to initiate the primary and secondary cracks. By analyzing Figure 6b, we observed that the failure order is analogous to the one related to static XFEM analysis, always with the exception of PET2na, in which damage initiation is predicted to happen after OP and PET. Moreover, all critical lacunae predicted in the fatigue analysis are related to damage initiation and progression even in static XFEM analysis (Table S2 of Supplementary Materials and Figure 6b).

**Figure 8:**
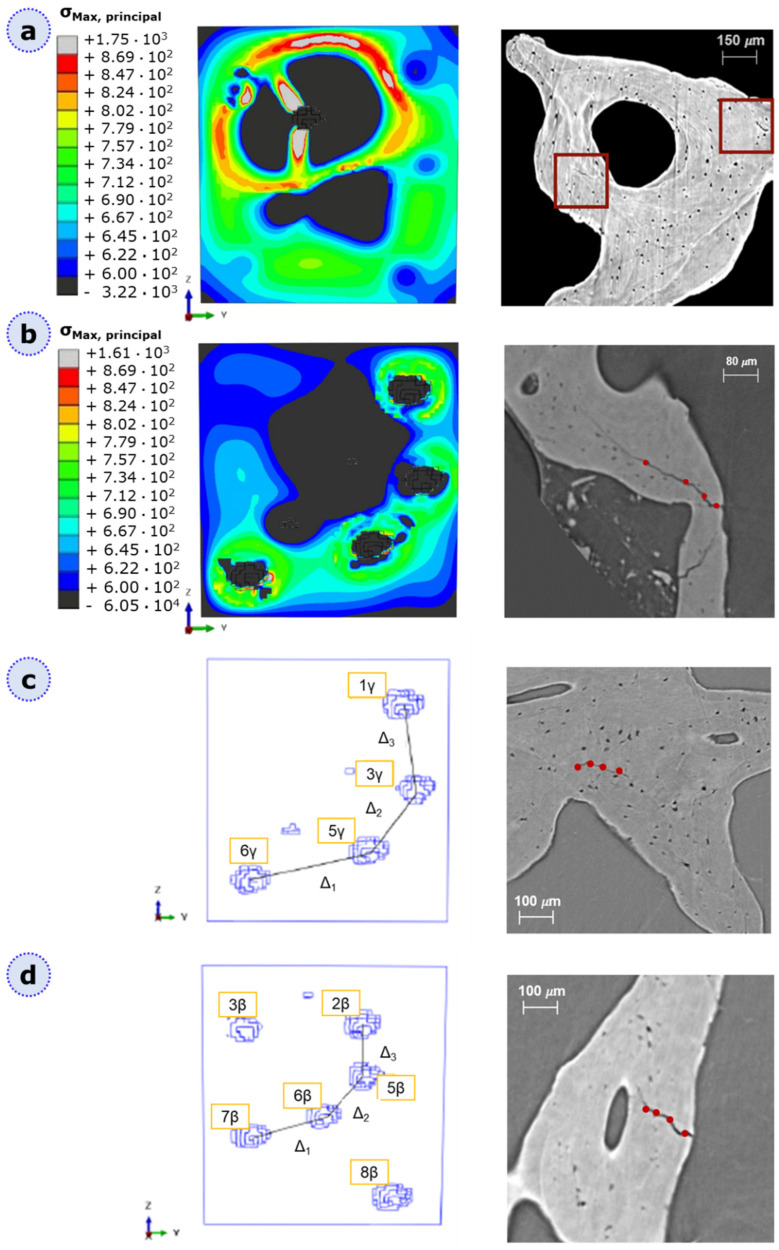
Parallelism in micro-crack pattern of AISI 316L bone-inspired geometries (left) and real bone (right). Bone images at micro-scale are obtained from synchrotron testing at 1.6 µm pixel size, which allows for visualization of the lacunar network. Synchrotron images are adapted with permission from [13] and obtained within the same research. Mechanical characteristics of bones are reported in [13]. (**a**) Lacunae have a weakening effect, acting as stress raisers (**b**) lacunae attract the crack, deviating its path. (**c**,**d**) Critical lacunar patters. Here, from the numerical side, we compared the inter-lacunar distances in different regions of the OP model, showing similar lacunar disposition but with different center-to-center distances, corresponding to Δ_1_, Δ_2_, Δ_3_, and Δ_4_. This pattern is present in PET and PETna too. The arrangement in (**c**) is related to the region near the traction surface, and the inter-lacunar distances are from Δ_1_ = 4.71 mm, Δ_2_ = 2.62 mm, and Δ_3_ = 2.86 mm. The (**d**) pattern is related to the β-region, 4 mm away from the traction surface; inter-lacunar distances, in this case, are Δ_1_ = 2.26 mm, Δ_1_ = 1.97 mm, and Δ_1_ = 1.69 mm.

By referring to damage progression patterns, we hypothesize that the most extended and interconnected damaged zones for each category correspond to the most probable fracture surfaces. No significant deviations from planar surfaces, whose normal is parallel to the loading axis, have been detected; it can be assumed that fracture of these geometries would occur under tensile opening mode I. We underline that this output is not fictitiously forced by the employment of specific computational parameters; on the contrary, the damage initiation criterion, MAXPS, was selected because it is a solution-dependent criterion. These lacunar arrangements could potentially lead to crack attraction sites (Figure 8a) and could also deviate from the crack path (Figure 8b, left).

By considering Figure 6b, OP and PET, all with twenty lacunae, face likely fracture at the same location: they tend to potentially break in the middle—4 mm from the traction surface—and they are characterized by the same lacunar disposition in that region (see Figure 8). Since the three models in question have different lacunar sizes and alignments, we believe that this disposition, with the centers of the lacunae belonging to the same ZY plane, is the most critical one, regardless of the morphological parameters and the distance from the traction surface. We can indeed discuss that in the remaining models, which are not characterized by this pattern, the predicted fracture plane lies elsewhere. We can highlight from Figure 8c,d on the left that a similar arrangement but with different inter-lacunar distances is actually present in the γ-region near the traction surface. However, this seems not to be crucial to model failure, mainly due to the higher inter-lacunar distances. Indeed, it is interesting that our models could be qualitatively compared with real bone micro-scale synchrotron images [13] (Figure 8), obtaining very similar crack patterns. This could be a prominent result, demonstrating that, independently from the material, lacunar voids play a role in fracture initiation and progression, and specific toughening lacunar patterns could be later exploited for practical biomedical applications.

## 5. Conclusions

In summary, our study provides a quantitative computational framework to investigate lacunae-micro-cracks existent interlinks by combining static XFEM and fatigue analyses. Furthermore, the work succeeds in demonstrating cross-talks between the lacunar network and damage initiation while highlighting the specific effect of both lacunar morphological and densitometric parameters on mechanical strength. An increase in lacunar density (as evidenced in OP2, PET2, and Pet2na), indeed, leads to a loss in mechanical strength al lower traction values, resulting as the most influencing parameter among the studied ones. Lacunar size (PET and Op categories), on the contrary, has a lower effect on mechanical strength, reducing it by 2%. Lacunar alignment (PET and PETna) has the main role of splitting the crack path.

Limitations could be linked to the reduced number of pores considered in the analysis, which is, however, linked to the significant computational power required to conduct XFEM analyses.

As future insights, we plan to realize the described morphologies via laser powder bed fusion using AISI 316L and later by exploiting other biomedical materials such as titanium. Since we have evidenced interesting toughening phenomena in our numerical analysis that are due to lacunar-like arrangements, we plan to translate these findings to the realization of biomedical products that could benefit from the lighter void-embedded geometry. The obtained results also indicate the potential of the developed approaches to shed some light on still obscure micro-damage phenomena when isolating micro-scale features as potential candidates for damage occurrence.

## Data Availability

Data are contained within the article.

## Supplementary Materials

The following supporting information can be downloaded at: https://www.mdpi.com/article/10.3390/ma16051931/s1: Table S1: Identification of the displacement at which the force peak occurs for all the considered geometries when using the lower DAF value; Table S2: Identification of lacunae that are sites of damage initiation in static-XFEM simulations and recap of the total number of lacunae involved in damage initiation for each category.
