# Supplementary material for: Isolating the Role of Bone Lacunar Morphology on Static and Fatigue Fracture Progression through Numerical Simulations
Source: Materials (Basel). 2023 Feb 26;16(5):1931. doi: 10.3390/ma16051931 (PMC10004234; doi:10.3390/ma16051931)
Supplement: Supplementary file 1 [file materials-16-01931-s001.zip › materials-2232963-supplementary.pdf]

materials

MDPI

Table S1. Identification of the displacement at which the force peak occurs for all the considered geometries when using the lower DAF value.

Category	Peak Force [N]	Displacement [mm]
OP	40,944	0.031
PET	44,985	0.033
PETna	45,637	0.034
PET2	46,681	0.034
OP2	48,067	0.035
PET2na	49,000	0.035

Table S2. Identification of lacunae that are sites of damage initiation in static-XFEM simulations and recap of the total number of lacunae involved in damage initiation for each category.

Category	α Region	β Region	γ Region	N° of Lacunae Involved by Damage
OP	2–4, 6	1–8	1, 3–6	17
OP2	-	2	3, 4	3
PET	1, 2	4–7	1, 3–5	10
PET2	-	3, 4	1, 3, 4	5
PETna	2–4	1, 4, 6	1-4	10
PET2na	-	-	5	1
